# Effect of atrial fibrillation on outcomes in patients with anterior circulation occlusion stroke receiving endovascular therapy

**DOI:** 10.3389/fnagi.2023.1160265

**Published:** 2023-06-15

**Authors:** Weijuan Wu, Jamir Pitton Rissardo, Thanh N. Nguyen, Mohammad Mofatteh, Hongquan Wei, David S. Liebeskind, Shuiquan Yang, Wanquan Li, Wanling Pan, Sijie Zhou, Yuzheng Lai, Jianfang Gao, Jian Wang, Ziqi Ouyang, Yuzhen Mai, Heng Meng, Yimin Chen, Xuxing Liao

**Affiliations:** ^1^Department of Neurology and Advanced National Stroke Center, Foshan Sanshui District People’s Hospital, Foshan, China; ^2^Department of Medicine, Federal University of Santa Maria, Santa Maria, Brazil; ^3^Department of Neurology, Radiology, Boston University Chobanian and Avedisian School of Medicine, Boston, MA, United States; ^4^School of Medicine, Dentistry and Biomedical Sciences, Queen’s University Belfast, Belfast, United Kingdom; ^5^Department of 120 Emergency Command Center, Foshan Sanshui District People’s Hospital, Foshan, Guangdong, China; ^6^UCLA Stroke Center and Department of Neurology, University of California, Los Angeles, Los Angeles, CA, United States; ^7^Department of Internal Medicine-Cardiovascular, Foshan Sanshui District People’s Hospital, Foshan, Guangdong, China; ^8^Department of Neurosurgery, First People’s Hospital of Foshan, Foshan, Guangdong, China; ^9^Department of Neurology, Guangdong Provincial Hospital of Integrated Traditional Chinese and Western Medicine (Nanhai District Hospital of Traditional Chinese Medicine of Foshan City), Foshan, Guangdong, China; ^10^Department of Research and Education, Foshan Sanshui District People’s Hospital, Foshan, China; ^11^Department of Neurosurgery and Advanced National Stroke Center, Foshan Sanshui District People’s Hospital, Foshan, Guangdong, China; ^12^Department of Neurology, Guangzhou Eighth People’s Hospital, Guangzhou Medical University, Guangzhou, Guangdong, China; ^13^Department of Neurology, The First Affiliated Hospital of Jinan University, Guangzhou, China; ^14^Neuro International Collaboration (NIC), Foshan, China

**Keywords:** cerebrovascular, atrial fibrillation, stroke, endovascular thrombectomy (EVT), patient outcome, occlusion

## Abstract

**Objective:**

Atrial fibrillation is one of the major risk factors of ischemic stroke. Endovascular thrombectomy (EVT) has become the standard treatment for acute ischemic stroke with large vessel occlusion. However, data regarding the impact of AF on the outcome of patients with acute ischemic stroke treated with mechanical thrombectomy are controversial. The aim of our study was to determine whether atrial fibrillation modifies the functional outcome of patients with anterior circulation acute ischemic stroke receiving EVT.

**Methods:**

We reviewed 273 eligible patients receiving EVT from January 2019 to January 2022 from 3 comprehensive Chinese stroke centers, of whom 221 patients were recruited. Demographics, clinical, radiological and treatment characteristics, safety outcomes, and functional outcomes were collected. Modified Rankin scale (mRS) score ≤ 2 at 90 days was defined as a good functional outcome.

**Results:**

In our cohort, 79 patients (35.74%) were eventually found to have AF. Patients with AF were elder (70.08 ± 11.72 vs. 61.82 ± 13.48 years, *p* = 0.000) and less likely to be males (54.43 vs. 73.94%, *p* = 0.03). The significant reperfusion rate (modified thrombolysis in cerebral infarction 2b-3) was 73.42 and 83.80% in patients with and without AF, respectively (*p* = 0.064). The good functional outcome (90-day modified Rankin scale: 0 to 2) rate was 39.24 and 44.37% in patients with and without AF, respectively (*p* = 0.460) after adjusting multiple confounding factors. There was no difference in the presence of symptomatic intracerebral hemorrhage between the two groups (10.13 vs. 12.68%, *p* = 0.573).

**Conclusion:**

Despite their older age, AF patients achieved similar outcomes as non-AF patients with anterior circulation occlusion treated with endovascular therapy.

## 1. Introduction

Atrial fibrillation (AF) is the most common non-structural cardiac condition related to ischemic cerebrovascular diseases (Junejo et al., 2020). One of the important risk factors associated with developing structural and non-structural cardiac abnormalities is aging (Yousufuddin and Young, 2019). With the worldwide population aging, the proportion of patients with AF is expected to rise in the coming decades (Kornej et al., 2020). A higher prevalence of AF in middle socio-demographic index countries and the in-hospital embolic recurrence in presumed cardioembolic stroke patients with AF cause a significant rise in medical cost (Arboix et al., 1998). Therefore, studies about the impact of AF on outcomes of thromboembolic stroke are of economic interest.

Atrial fibrillation is associated with poor functional outcomes and increased hemorrhagic complications after intravenous thrombolysis (IVT) (Kimura et al., 2009; Sanak et al., 2010). Bridging IVT improved functional outcomes in non-AF patients, but the benefits did not extend to AF patients (Akbik et al., 2022; Loo et al., 2023). However, the impact of AF on the treatment effect of mechanical thrombectomy (MT) remains controversial. Patients with AF responded significantly better to MT than those without AF did (Lin et al., 2020), while other studies showed that AF experienced worse 90-day outcomes, even in the setting of similar rates of successful reperfusion and independent negative predictors of good long-term functional outcome (Zdraljevic et al., 2022; Kobeissi et al., 2023). In addition, age > / = 80 years was a significant predictor of unfavorable outcomes after EVT for AIS patients with AF (Jiao et al., 2022).

We performed a retrospective study based on a multicenter stroke registry to compare the technical and functional outcomes in acute ischemic stroke patients with and without AF after receiving EVT. We also evaluated the impact of age in AF patients with anterior circulation occlusion after EVT.

## 2. Patients and methods

### 2.1. Study population

A hospital-based cohort of 273 subjects who underwent EVT was recruited from three comprehensive stroke centers in Foshan, China from January 2019 to January 2022. The study was approved by the Ethical Board of the Foshan Sanshui District People’s Hospital in China. Medical records were assessed for data on socio-demographic characteristics, clinical features, neuroimaging, and EVT results.

The inclusion criteria for this study were: (1) age ≥18, (2) independent daily living [modified Rankin scale (mRS < 3)] before the index stroke, (3) undergoing EVT within 24 h of symptom onset, (4) presence of intracranial internal carotid artery (ICA) and/or anterior circulations occlusions M1 (MCA-M1) and M2 (MCA-M2) segments of the middle cerebral artery (MCA), or tandem occlusion. Exclusion criteria were: (1) lost to follow-up, (2) other vessel occlusions. Screening and treating AF are two important aspects of managing AF. 12-lead electrocardiogram (ECG) was used for all patients in our hospital, and 24-h ECG (Holter) monitoring was used for all cardiac embolic stroke subgroups. However, post-stroke in-hospital rhythm monitoring was limited by a finite window of observation, which was particularly problematic in the context of intermittent AF. For all AF patients, anticoagulants were used when CT exclude hemorrhage after EVT according to the consensus rule of “1-3-6-12” days. Oral anticoagulants included warfarin and target-specific anticoagulants, also known as direct oral anticoagulants. We usually use the TOAST (Trial of Org 10172 in Acute Stroke Treatment), which includes large-artery atherosclerosis (LAA), cardioembolism (CE), small-artery occlusion lacunar (SAA), stroke of other determined etiology (SOE), and stroke of undetermined etiology (SUE). However, subjected to the condition in the primary hospital, the non-invasive examination was particularly problematic in the screening of patients with intermittent AF, thereby compromising the accuracy of CE, SOE, and SUE.

### 2.2. Data collection and measurements

Patients’ demographic data (age, sex), comorbidities (AF, hypertension, diabetes mellitus, coronary artery diseases, hyperlipidemia, chronic kidney disease, smoking history, past history of stroke), laboratory tests (triglyceride, low-density lipoprotein, high-density lipoprotein, total cholesterol, and glucose at admission) were recorded. Stroke severity [National Institute of Health Stroke Scale/Score (NIHSS) score at admission], time intervals (door-to-puncture, door-to-recanalization time, last-known-well-to-puncture time, door-to-puncture time, puncture to recanalization time), functional outcomes (NIHSS score after EVT and mRS), and symptomatic intracranial hemorrhage (sICH) were assessed. A favorable functional outcome was defined as mRS 0 to 2 at 90-day follow-up (Yang et al., 2022).

### 2.3. EVT procedures

The decision to perform EVT was made by the team of vascular neurologists and treating neuroradiologists. Board-certified interventional neuroradiologists performed the EVT procedure. In eligible patients, EVT was performed according to the European Stroke Organization guideline by the European Society for Minimally Invasive Neurological Therapy guidelines (Turc et al., 2019). Mechanical thrombectomy was performed from femoral puncture using a guiding sheath and an intermediate catheter and stent retriever. Substantial reperfusion was defined as modified Thrombolysis in Cerebral Infarction scale (mTICI) 2b or 3 at the completion of the procedure (Chen et al., 2022). Patients were admitted to the stroke unit for close monitoring and observation for at least 24 h postoperatively.

### 2.4. Statistical analysis

Double data entry and standardized procedures were performed for quality checking. No imputations were made for missing data. The data were then transported to the IBM SPSS software version 26 (IBM Corp., Armonk, NY) for statistical analysis. The Kolmogorov-Smirnov test was used for assessing normality. Categorical variables were represented as proportions, and continuous variables were described as means and standard deviations. A *p*-value < 0.05 was considered significant in all analyses. As appropriate, we used an independent Student’s *t*-test and Mann–Whitney U test to compare basic characteristics between groups. The categorical variables were analyzed using Chi-squared (χ^2^) or Fisher’s exact tests if the expected number was ≤5. We created unadjusted and adjusted binary logistic regression models to determine associations between EVT and the study outcomes adjusted for age and other variables recorded.

## 3. Results

A total of 273 subjects were eligible for the study. Three patients were lost to follow-up due to non-compliance; one patient was excluded because of presentation with occlusion of the M3 segment of the MCA; 40 subjects were excluded due to basilar artery occlusion; eight subjects had other multiple vessel occlusions. 221 patients (79 with AF, 142 without AF) were included in the final study. Table 1 summarizes the characteristics of the study participants.

**TABLE 1:** Comparison of baseline characteristics in patients with and without atrial fibrillation.

	AF group	No AF group	*P*
Number	79	142	
Age mean ± SD	70.08 ± 11.72	61.82 ± 13.48	0.000
Male (n %)	43 (54.43%)	105 (73.94%)	0.003
Hypertension (n %)	48 (60.76%)	80 (56.34%)	0.523
Diabetes mellitus (n %)	16 (20.25%)	32 (22.54%)	0.693
Coronary artery disease (n %)	18 (22.78%)	21 (14.79%)	0.135
Prior stroke (n %)	16 (20.25%)	19 (13.38%)	0.180
Hyperlipidemia (n %)	15 (18.99%)	22 (15.49%)	0.505
Chronic kidney disease (n %)	12 (15.19%)	10 (7.04%)	0.053
Current Smoker (n %)	7 (8.86%)	35 (24.65%)	0.004
NIHSS Pre-EVT (IQR)	16.00 (12.00, 20.00)	14.00 (10.75, 17.00)	0.017
ASPECTS pre-treatment (IQR)	8.00 (8.00, 9.00)	8.00 (8.00, 9.00)	0.496
mRS before stroke (IQR)	0.00 (0.00, 0.00)	0.00 (0.00, 0.00)	0.059
ICA (n %)	20 (25.32%)	35 (24.65%)	0.957
MCA-M1 (n %)	38 (48.10%)	67 (47.18%)	
MCA-M2 (n %)	6 (7.59%)	14 (9.86%)	
Tandem (n %)	15 (18.99%)	26 (18.31%)	
IV thrombolysis (n %)	40 (50.63%)	52 (36.62%)	0.043
Door-to-puncture time (IQR), min	146.00 (105.00, 190.00)	142.50 (109.75, 190.75)	0.947
Door-to-recanalization time (IQR), min	225.00 (164.00, 280.00)	234.50 (177.75, 309.75)	0.209
puncture to recanalization time (IQR), min	57.00 (38.00, 100.00)	70.00 (46.75, 114.00)	0.037
Last-known-normal to puncture time (IQR), min	257.00 (193.00, 360.00)	309.00 (193.75, 511.25)	0.136

AF, atrial fibrillation; IQR, interquartile range; IV, intravenous; NIHSS, National Institute of Health Stroke Scale.

Comparing the AF group and no AF group, the AF group was older (70.08 ± 11.72 vs. 61.82 ± 13.48 years old, *p* < 0.001), had fewer male sex [54.43 vs. 73.94% (*p* = 0.003)], had fewer smokers (8.86 vs. 24.65%, *p* = 0.004), had higher median NIHSS Pre-EVT (16 vs. 14, *p* = 0.017), underwent more IV thrombolysis before EVT [50.63 vs. 36.62% (*p* = 0.043)], but had a shorter median puncture to recanalization time (57 vs. 70 min, *p* = 0.037). The other baseline characteristics between patients with and without AF did not differ significantly.

**FIGURE 1:**
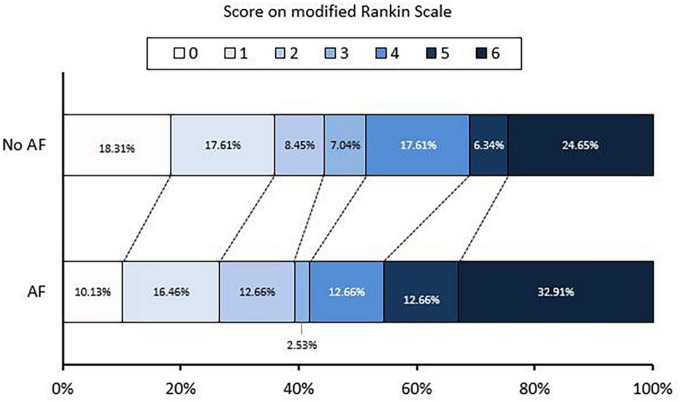
Distribution of 3-month mRS of No AF and AF groups.

In unadjusted analyses, individuals undergoing EVT with AF compared to those without AF did not present any difference in favorable functional outcomes (90-day outcome 39.24 vs. 44.37%, *p* < 0.460) (Table 2). Figure 1 depicts the distribution of 90-day mRS in both groups.

**TABLE 2:** Comparison of outcome of AF and No AF group.

	AF	No AF	X^2^/z	*P*
mTICI 2b-3	58 (73.42%)	119 (83.80%)	3.433	0.064
mTICI 3	28 (35.44%)	55 (38.73%)	0.234	0.628
No. of passes (IQR)	1.00 (1.00, 3.00)	2.00 (1.00,3.00)	−1.215	0.224
sICH (n %)	8 (10.13%)	18 (12.68%)	0.318	0.573
24 h NIHSS	15.00 (6.00, 20.00)	12.00 (5.00, 18.00)	−1.707	0.088
Length of stay	11.00 (6.00, 25.00)	12.00 (7.00, 18.25)	−0.046	0.963
mRS discharge (IQR)	4.00 (2.00, 5.00)	4.00 (1.00, 5.00)	−1.720	0.085
90-day favorable outcome	31 (39.24%)	63 (44.37%)	0.546	0.460
90-day mortality	26 (32.91%)	35 (24.65%)	1.735	0.188

mTICI, modified thrombolysis in cerebral infarction; IQR, interquartile range; NIHSS, National Institute of Health Stroke Scale; sICH, symptomatic intracranial hemorrhage. Favorable outcome: mRS (0–2) at 3 months. Poor outcome: mRS ≥ 3.

In the binary logistic regression model, we found persistence of the findings of the comparison between the 90-day outcome of AF and non-AF individuals after controlling for other factors. Table 3 provides results of 90-day outcome (*p* = 0.629) and 90-day mortality (*p* = 0.802) after adjusting for age (<65 years), which were not significant.

**TABLE 3:** Comparison of 90-day outcome (AF group vs. non-AF group) after adjusting for age.

	OR	95% CI	*P*
90-day favorable outcome	1.162	0.633–2.133	0.629
90-day mortality	1.086	0.570–2.069	0.802

In Table 4, age, male sex, CKD, current smoking history, and mRS before stroke were controlled. Similar to previous findings, there were no statistically significant differences for 90-day outcome (*p* = 0.482) and 90-day mortality (*p* = 0.928).

**TABLE 4:** Comparison of 90-day outcome (AF group vs. non-AF group) after adjusting age, male sex, chronic kidney disease, current smoking history, and mRS before stroke.

	OR	95% CI	*P*
90- -day favorable outcome	1.257	0.664–2.381	0.482
90-day mortality	1.032	0.518–2.058	0.928

In the last model (Table 5), the variables controlled were age, male sex, chronic kidney disease, current smoking status, NIHSS Pre-EVT, mRS pre-treatment, intravenous thrombolysis, last-known-well-to-puncture time, and puncture to recanalization time. After such controls, no statistically significant differences were found for 90-day outcome (*p* = 0.461) and 90-day mortality (*p* = 0.661).

**TABLE 5:** Comparison of 90-day outcome (AF group vs. non-AF group) after adjusting age, male sex, chronic kidney disease, current smoking history, NIHSS Pre-EVT, mRS pre-treatment, IV thrombolysis, last-known-well-to-puncture time, and puncture to recanalization time.

	OR	95% CI	*P*
90-day favorable outcome	1.300	0.647–2.612	0.461
90-day mortality	0.838	0.381–1.845	0.661

## 4. Discussion

This retrospective cohort study provides insights into the outcomes of patients with AF and without AF who underwent EVT for anterior circulation occlusion at three comprehensive stroke centers in China. Our study showed no differences in early and long-term favorable outcomes between the two groups of patients.

Previous studies assessing complications related to EVT reported that AF was a significant risk factor for complications, especially intracranial hemorrhage. A retrospective analysis of consecutive patients presenting to 13 high-volume stroke centers revealed that AF compared to no-AF individuals with anterior circulation occlusion, had a significant risk of parenchymal hematomas [OR 1.61 (1.01–2.55), *p* < 0.045] (Nogueira et al., 2015). More recent cohort studies revealed that patients with AF and no-AF patients had better endovascular mechanical thrombectomy outcomes (90-day favorable outcome 55.8 vs. 17.5%, *p* < 0.01); however, this benefit was not persistent in elderly patients (>70 years) (Lin et al., 2020). In the present cohort, age did not affect the endovascular outcome in patients with or without AF.

We believe that the positive outcomes encountered in the present study can be attributed to two main factors. First, the development of the endovascular technique and devices probably influenced the outcomes. Second, the present cohort only included individuals with anterior circulation proximal LVO. In other studies, different territories were assessed, and a sub-analysis of their findings was later conducted.

A recent study by Zdraljevic et al. (2022) found that AF is an independent negative predictor of good long-term functional outcome (OR 0.29, 95% CI 0.11–0.78, *p* = 0.01). Therefore, there is a possible bias regarding the EVT procedure technique and selection bias. Moreover, another multicenter retrospective study assessing the clinical outcomes of 219 individuals with valvular versus non-valvular AF in acute anterior circulation occlusive stroke undergoing EVT showed that both groups had similar safety and functional outcomes (Li et al., 2019). The authors explained that chronic heart failure should be evaluated in stroke studies because this condition confers three times the risk of poor functional outcomes.

Furthermore, a Medicare study extracted data from 4,627 subjects who underwent EVT. In the unmatched cohorts, patients with AF were older and had a significantly greater burden of key comorbidities than the non-AF group (age 74 y ± 11 vs. 60 y ± 15, *p* < 0.0001) (Munir et al., 2017). However, there was no difference between the groups when mortality, morbidity, and even costs were evaluated (Munir et al., 2017).

Recently, a systematic review and meta-analysis including 10 studies with 6,543 patients conducted by Kobeissi et al. (2023) found that there were comparable rates of mRS scores of 0 to 2 between patients with AF and patients without AF (odds ratio [OR], 0.72 [95% CI, 0.47–1.10]; *P* = 0.13), with significant heterogeneity (*I*^2^ = 75%; *P* < 0.001) among the included studies. After sensitivity analysis, the rate of mRS scores of 0 to 2 was significantly lower among patients with AF (OR, 0.65 [95% CI, 0.52–0.81]; *P* < 0.001), with heterogeneity (*I*^2^ = 55%; *P* = 0.02). Noteworthy, there is significant heterogeneity among the included studies in Kobeissi et al.’s study. Heterogeneity was assessed using the Q statistic and the I^2^ test, in which I^2^ greater than 50% or *P* < 0.05 were considered significant. Therefore, we can conclude that further studies are required to examine the conclusion of Kobeissi et al. (2023). Additionally, the heterogeneity in the results may partially be attributed to the higher rates of diabetes and hypertension in the AF group in Kobeissi et al.’s study. An important factor that can modify the outcomes of patients undergoing EVT is the previous use of intravenous thrombolysis. Yaghi et al. (2020) assessed intravenous alteplase and its effects in AF patients with cardioembolic stroke. The authors revealed that thrombolysis could reduce the 90-day mortality of patients with acute ischemic stroke with AF not undergoing EVT (OR, 0.58; 95% CI, 0.39–0.87).

Other studies described age as an important risk factor for worse outcomes related to anterior circulation stroke after EVT (Castonguay et al., 2014; Lasek-Bal et al., 2022). However, in the HERMES meta-analysis, the subgroup of patients who were older still benefited significantly from EVT (Goyal et al., 2016). In this context, our study described the modeling as a determinant variable of 65 years, and favorable outcomes were encountered (Yousufuddin and Young, 2019). We believe that more studies regarding the definition of this variable as 65 years should be conducted to explain the influence of this independent variable better.

Globally, the population aged 65 and over is growing faster than all other age groups. Concurrently, there is a significant increase in cardiovascular risk factors and the development of atrial fibrillation. Therefore, studies assessing the influence of age on outcomes are essential for prognostic risk stratification. Moreover, studies of specific age groups of the population can lead to the development of new techniques and other practices promoting a favorable outcome.

Limitations of our study include the relatively small size of our sample. Secondly, our cohort was hospital-based and not population-based, which could have biased our results. Thirdly, the retrospective nature of this study may have resulted in information bias. Fourthly, we cannot exclude the introduction of bias caused by the different routines of each physician. Also, it is worth mentioning that no histopathological differentiation was done between red thrombi (red blood cell-rich) and white thrombi (fibrin-rich). Despite such limitations, the current study can highlight the significance of functional outcome after EVT in patients with AF who have anterior circular occlusion.

In conclusion, after adjusting for confounding factors such as age, male sex, chronic kidney disease, current smoking history, NIHSS Pre-EVT, mRS pre-treatment, IV thrombolysis, last-known-well-to-puncture time, and puncture to recanalization time, AF patients achieved similar outcomes as non-AF patients with anterior circulation occlusion treated with endovascular therapy.

## Data availability statement

The raw data supporting the conclusions of this article will be made available by the authors, without undue reservation.

## Ethics statement

The study was reviewed and approved by the Medical Ethical Committees of the Foshan Sanshui District People’s Hospital. Written informed consent from the participants’ legal guardians/next of kin was not required to participate in this study per the national legislation and institutional requirements. Written informed consent for participation was not required for this study in accordance with the national legislation and the institutional requirements.

## Author contributions

YC, WW, JP, HM, SY, and XL conceived the idea and drafted the manuscript. TN, MM, and DL provided critical revision. WL, WP, SZ, YL, JG, JW, ZO, and HW collected data and reviewed the manuscript. All authors contributed to the manuscript and read and approved the final version of the manuscript.
